# Conditional knockout of N-WASP in mouse fibroblast caused keratinocyte hyper proliferation and enhanced wound closure

**DOI:** 10.1038/srep38109

**Published:** 2016-12-02

**Authors:** Neeraj Jain, Pazhanichamy Kalailingam, Kai Wei Tan, Hui Bing Tan, Ming Keat Sng, Jeremy Soon Kiat Chan, Nguan Soon Tan, Thirumaran Thanabalu

**Affiliations:** 1 School of Biological Sciences, Nanyang Technological University, 60 Nanyang Drive, Singapore 637551, Republic of Singapore; 2 Institute of Molecular and Cell Biology, 61 Biopolis Drive, Proteos, Agency for Science Technology & Research, 138673, Singapore; 3 KK Research Centre, KK Women’s and Children’s Hospital, 100 Bukit Timah Road, 229899, Singapore

## Abstract

Neural-Wiskott Aldrich Syndrome Protein (N-WASP) is expressed ubiquitously, regulates actin polymerization and is essential during mouse development. We have previously shown that N-WASP is critical for cell-ECM adhesion in fibroblasts. To characterize the role of N-WASP in fibroblast for skin development, we generated a conditional knockout mouse model in which fibroblast N-WASP was ablated using the Cre recombinase driven by Fibroblast Specific Protein promoter (Fsp-Cre). N-WASP^FKO^ (*N-WASP*^*fl/fl*^*; Fsp-cre*) were born following Mendelian genetics, survived without any visible abnormalities for more than 1 year and were sexually reproductive, suggesting that expression of N-WASP in fibroblast is not critical for survival under laboratory conditions. Histological sections of N-WASP^FKO^ mice skin (13 weeks old) showed thicker epidermis with higher percentage of cells staining for proliferation marker (PCNA), suggesting that N-WASP deficient fibroblasts promote keratinocyte proliferation. N-WASP^FKO^ mice skin had elevated collagen content, elevated expression of FGF7 (keratinocyte growth factor) and TGFβ signaling proteins. Wound healing was faster in N-WASP^FKO^ mice compared to control mice and N-WASP deficient fibroblasts were found to have enhanced collagen gel contraction properties. These results suggest that N-WASP deficiency in fibroblasts improves wound healing by growth factor-mediated enhancement of keratinocyte proliferation and increased wound contraction in mice.

The skin is the largest organ in the body and is composed of two primary layers; the epidermis and the dermis which are separated by a basement membrane^1^. The epidermis is composed mainly of keratinocytes whereas the dermis is composed mainly of connective tissue produced by dermal fibroblasts. As a protective organ, the skin requires mechanical toughness and the ability to repair itself after wounding. Wound healing can be broken down to 3 stages, the inflammatory phase, proliferation phase and the maturation phase. During the inflammatory phase, the activated platelets play an important role in the formation of a clot to stop bleeding and maintain tissue homeostasis. Both the keratinocyte and fibroblasts play a critical role during the proliferation phase. The keratinocytes proliferate and migrate to re-epithelize the epidermis layer and cover the wound while underlying fibroblasts migrate and is activated to synthesize extracellular matrix proteins for connective tissue^2^,^3^. Upon tissue injury, fibroblasts differentiate into myofibroblasts and promote wound healing by decreasing the size of the wound in mice. Myofibroblasts express highly contractile protein α-smooth muscle actin (α-SMA) which provides adhesive and biochemical force required for wound closure^4^,^5^. Several studies have shown the importance of fibroblasts in wound healing. Rac1 knockout in dermal fibroblast led to delay in wound healing, reduced myofibroblast activation and lowered collagen production^6^. Fibroblast specific deletion of TGFβ receptor diminished the wound contraction, dermal regeneration and re-epithelialization^7^. Similarly, reduced TGFβ signaling was identified in fibroblast specific integrin β1 deficient mice^8^. All these studies suggest that the dermal fibroblast is critical for *in-vivo* tissue repair.

Keratinocytes and fibroblasts communicate through the secretion of growth factors and cytokine^9^. During wound healing, fibroblasts within the wound bed secrete keratinocyte growth factor (KGF) that triggers keratinocytes to proliferate, facilitating re-epithelization^10^. Moreover, exogenous KGF-1 expression into the excisional wounds of genetic diabetic mouse models, improved their wound healing rate and epithelialization^11^. 12-O-tetradecanoylphorbol-13-acetate (TPA) induced epithelial cells proliferation and local inflammation was found to be reduced in the absence of proliferating fibroblast^12^. In short, fibroblasts play an important role in the secretion of growth factors required for keratinocyte proliferation during the skin wound healing process.

Cell migration, adhesion, morphology, growth factor secretion, proliferation and other critical cellular process are regulated by the actin cytoskeleton^13^. N-WASP (Neural-Wiskott Aldrich Syndrome protein) which is expressed ubiquitously and has been shown to be a critical regulator of actin cytoskeleton remodeling via Arp2/3 complex^14^. N-WASP deficiency in fibroblast leads to reduced cell-ECM adhesion and increased cell motility^15^. N-WASP regulates invadopodia formation^16^ in Src transformed fibroblasts^17^,^18^ and affects clathrin dependent^19^ or independent endocytosis^20^. Moreover, N-WASP has been found to play critical role in RNA polymerase-II dependent transcription regulation, RNA processing and DNA repair via interaction with PSF-NonO complex^21^. N-WASP expression has been found to be lower in breast cancer cells^22^, whereas it highly expressed in hepatocellular carcinoma^23^.

N-WASP deficient mice had abnormalities in neural tube formation, heart and mesoderm differentiation and die at embryonic stage E11.5^24^,^25^. Conditional knockout of N-WASP expression in mouse brain leads to severe hydrocephalus condition and premature post-natal death^26^. N-WASP is also critical for T-cells development^27^. Conditional ablation of N-WASP expression in keratinocytes leads to ulceration, alopecia and marked epidermal hyperproliferation. This disrupted hair follicle cycling was due to increased TGFβ signaling^28^ or due to decreased Wnt-dependent transcription^29^.

In this study we have characterized the *in-vivo* role of N-WASP in dermal fibroblasts using Fsp-Cre (fibroblast specific protein) mice to selectively ablate the expression of N-WASP in fibroblasts. N-WASP^fl/fl^; Fsp-cre (N-WASP^FKO^) mice were born following Mendelian genetics suggesting that deficiency of N-WASP in fibroblast does not lead to lethality. Both the male and female N-WASP^FKO^ mice were sexually active and did not show any physical abnormalities. Hematoxylin and eosin staining of skin sections of the N-WASP^FKO^ mice showed thicker epidermis suggesting that N-WASP deficiency in dermal fibroblasts leads to epidermal hyperproliferation in aged mice. Moreover, the dermal wound closure was found to be faster in N-WASP^FKO^ mice compared with the controls. *In-vitro* collagen gel contraction assay showed that N-WASP deficient fibroblast caused faster collagen gel contraction in the presence of TGFβ. Expression of growth factor KGF/FGF7 was elevated in N-WASP^FKO^ mice. In addition the expression of TGFβ signaling pathway proteins (Smad2, Smad3, Tak1 and TGFβ-RII) was increased in N-WASP^FKO^ mice compared to control. Thus, our results suggest that the ablation of N-WASP expression in dermal fibroblasts enhanced wound healing, in part, by promoting proliferation of keratinocytes and enhanced wound contraction. Our data provide new insights into the functions of N-WASP in dermal fibroblasts regulating epidermal cell proliferation.

## Results

### Loss of N-WASP expression in dermal fibroblasts caused epidermal hyper proliferation in aged mice

**Figure 1:**
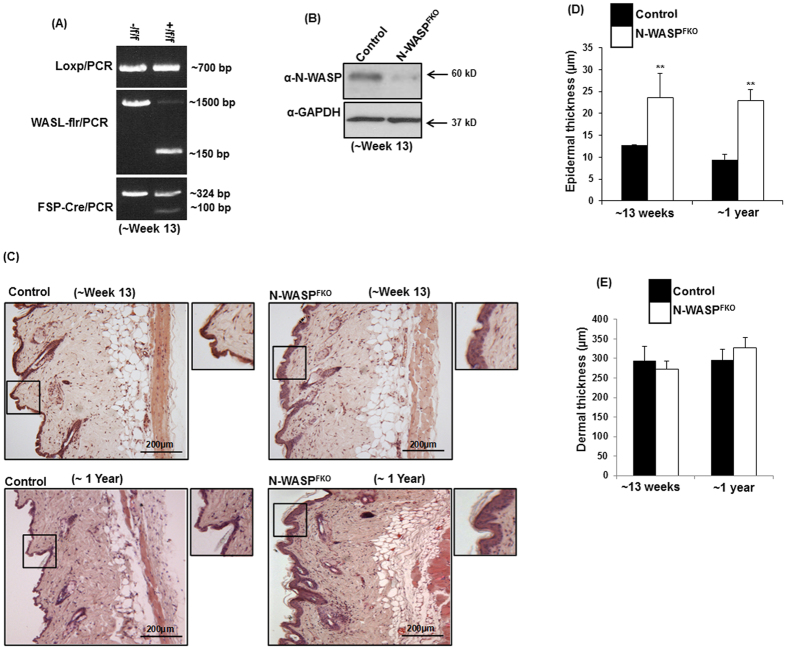
Enhanced epidermal thickness in N-WASP^FKO^ mice. (**A**) Genomic PCR analysis of deletion of N-WASP exon 3 and 4 in dermal fibroblasts cells isolated from control and N-WASP^FKO^ mice. (**B**) Western blot analysis for N-WASP expression of dermal fibroblasts cells isolated 13 weeks old mice. (Note: we achieved greater than 80% pure fibroblasts population from our mice skin fibroblasts isolation method, it contain a little population of other cell types as well). (**C**) H&E stained dorsal skin sections from 13 weeks and 1 year old mice. Quantification of epidermal (**D**) and dermal (**E**) thickness of control and N-WASP^FKO^ mice skin sections. N-WASP^FKO^ mice have more than two epidermal layers compared with control mice. **Represent *p* < 0.01.

**Figure 2:**
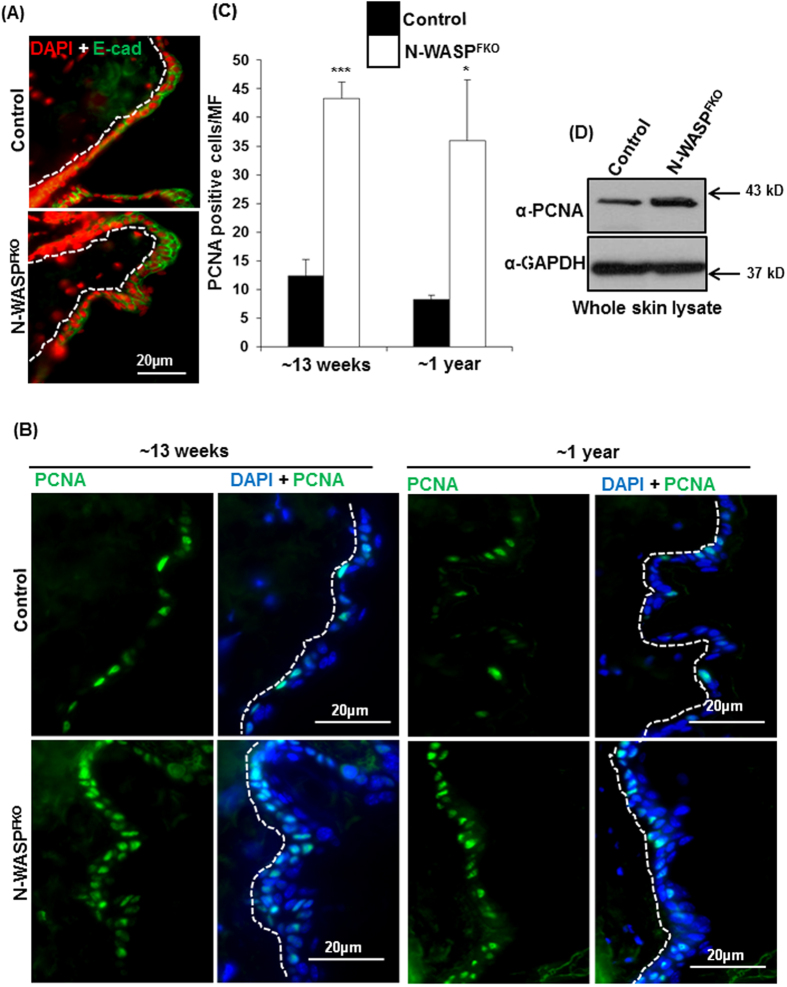
N-WASP deficiency in dermal fibroblast enhanced epidermal cell proliferation. (**A**) Distribution of E-cadherin in control and N-WASP^FKO^ mice was normal but N-WASP^FKO^ mice showed multiple layers of epidermal cells. Red: DAPI (**B**) Epidermal proliferation measured by PCNA immuno-staining of paraffin embedded dorsal skin sections of 13 weeks and 1 year old control and N-WASP^FKO^ mice. Blue: DAPI (**C**) Increased number of PCNA positive cells/MF was identified by quantification in N-WASP^FKO^ mice. *Represent *p* < 0.05; ***p < 0.001. (**D**) Western blot analysis for PCNA from whole skin lysate of one year old control and N-WASP^FKO^ mice. N-WASP^FKO^ mice showed enhanced PCNA expression in skin compared with control mice.

In this study, we have generated dermal fibroblast specific N-WASP knockout mice by crossing *N-WASP*^*fl/fl*^mice with Fsp-cre mice (Fig. S1A). The heterozygous, *N-WASP*^*fl/WT*^*; Fsp-cre* mice from the first generation mice were back crossed to get *N-WASP*^*fl/fl*^*; Fsp-cre* (N-WASP^FKO^) mice near to the Mendelian 25% ratio (Fig. S1B). Excision of exon 3 and 4 of (floxed) N-WASP gene was detected in N-WASP^FKO^ mice (Fig. S1C) in tail genomic PCR. Primary dermal fibroblasts isolated from N-WASP^FKO^ mice showed efficient knockdown of N-WASP expression at 13 weeks after birth (Fig. 1A,B). N-WASP^FKO^ mice exhibited a normal life span and no obvious defects were observed in body weight (Fig. S1D), external morphology, reproductive vigor and behavior compared to control littermates (Data not shown). N-WASP expression in keratinocytes has been shown to play critical role in hair follicle cycling via the anti-proliferative TGFβ pathway^28^. Histological analysis of skin layers of N-WASP^FKO^ mice did not show any obvious differences in hair follicle pattern or numbers between weeks 4 to 6 after birth (Fig. S2A). Histological analysis of mice skin taken after 13 weeks or nearly 1 year after birth showed a significant increase in epidermal layer thickness in N-WASP^FKO^ mice compared to either heterozygous (*N-WASP*^*fl/WT*^*; Fsp-cre)* or control (*N-WASP*^*fl/fl*^) mice (Fig. 1C,D S2B). There was no significant difference in the dermal thickness of mice skin (Fig. 1E). In addition, histological analysis of the ear also showed an enhanced thickening in the N-WASP^FKO^ mice, compared to the control littermates (Data not shown). The thickening of the epidermal layer of N-WASP^FKO^ mice was further characterized by visualizing the nucleus and E-cadherin structures which showed the presence of more than two layers of cells in the epidermis. There was no obvious alteration in the epidermal cell-cell junction, distribution of E-cadherin between control and N-WASP^FKO^ mice (Fig. 2A). In order to verify that the thickening in epidermal layer in N-WASP^FKO^ mice skin is due to a proliferative defect within the epidermis, we performed the immuno-staining of paraffin embedded skin sections for the proliferation marker PCNA. Basal level of PCNA positive cells count revealed that N-WASP^FKO^ mice has significantly higher (40 ± 5, 13 weeks; 36 ± 10, 1-year-old) number of PCNA positive keratinocytes compared with control mice (12 ± 3, 13 weeks; 8 ± 1, 1-year-old) (Fig. 2B,C). Furthermore, Western blot analysis of whole skin lysate showed higher expression of PCNA in N-WASP^FKO^ mice (Fig. 2D). Thus, suggesting that N-WASP^FKO^ mice have defects in regulating the proliferation of keratinocytes resulting in epidermal hyperproliferation.

### N-WASP^FKO^ mice do not have defect in skin barrier function

**Figure 3:**
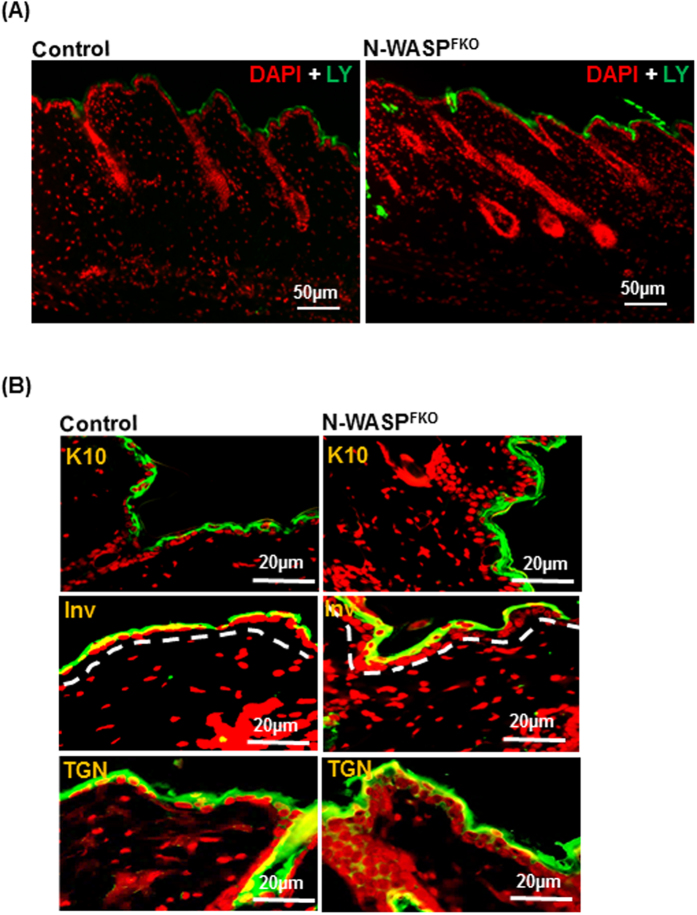
N-WASP deletion does not affect skin barrier function. (**A**) Small freshly isolated sections of skin were analyzed for Lucifer yellow penetration assay. The permeability barrier function was not affected in N-WASP^FKO^ mice. (**B**) 13 weeks old control and N-WASP^FKO^ mice skin were stained with early (keratin 10, transglutaminase) and terminal (involcurin) differentiation markers. There were no obvious differences in expression of differentiation markers observed between control and N-WASP^FKO^ mice. Red: DAPI.

In order to analyze the skin barrier function we isolated a section of skin from control and N-WASP^FKO^ mice and incubated with Lucifer yellow on the epidermis. The skin section was subsequently analyzed by histological staining to visualize the presence of Lucifer yellow using fluorescent microscopy. In both control and N-WASP^FKO^, fluorescent dye was restricted to the outer layer of epidermis suggesting that there was no obvious defect in the skin barrier function of the epidermis in N-WASP^FKO^ mice (Fig. 3A). The differentiation of keratinocytes in N-WASP^FKO^ and control mice was analyzed by immunofluorescence staining for early and terminal differentiation markers (keratin 10, involucrin, transglutaminase). There were no significant differences in the staining pattern of the differentiation markers between control and N-WASP^FKO^ mice, however there was a thicker layer of cells staining for keratin 10, involucrin and transglutaminase. This suggests that N-WASP deletion in dermal fibroblast cells do not alter epidermal cells differentiation (Fig. 3B).

### TPA induced hyperproliferation response

**Figure 4:**
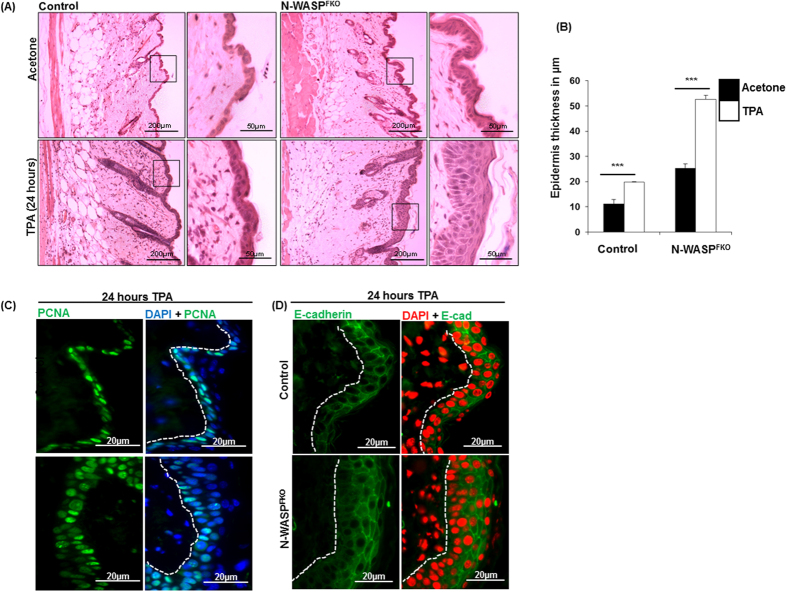
TPA induced hyperproliferation in N-WASP^FKO^ mice. (**A**) Histological analysis of solvent (acetone) and TPA treated (for 24 hours) dorsal skin of control and N-WASP^FKO^ mice by H & E staining. (**B**) Quantification of epidermal thickness of acetone and TPA treated dorsal mice skin. N-WASP^FKO^ mice skin is significantly thicker than control mice skin after TPA treatment. (**C**) Immuno-staining of TPA treated mice skin for analysis of PCNA positive cells. N-WASP^FKO^ mice skin showed significant more number of PCNA positive epidermal cells compared with control mice skin. (**D**) Analysis of cell-cell junctions by E-cadherin staining in TPA treated mice.

In order to investigate the cutaneous response under the stress condition, we treated the skin of control and N-WASP^FKO^ mice topically with the phorbol ester, 12-O-tetradecanoylphorbol-13-acetate (TPA). Expectedly, a single treatment of 6.5 nM/50 μl TPA for 24 hours on dorsal skin resulted in significant thickening of epidermis of both control and N-WASP^FKO^ mice compared with vehicle-treated mice (Fig. 4A,B). Histological analyses of TPA-treated skin sections revealed an elevated hyperproliferative effect in N-WASP^FKO^ mice skin, compared to wildtype littermates. (Fig. 4A), with a concomitant increase in the number of PCNA-positive proliferating keratinocytes in N-WASP^FKO^ mice skin (Fig. 4C). There was no alteration in the epidermal cell-cell junctions identified in stressed induced (TPA-treated) N-WASP^FKO^ mice skin as localization and pattern of E-cadherin was comparable to TPA treated control mice skin (Fig. 4D). Thus, TPA treatment led to enhanced epidermal cell hyperproliferation in N-WASP^FKO^ mice compared to control mice.

### Loss of N-WASP expression in dermal fibroblast results in faster wound closure

**Figure 5:**
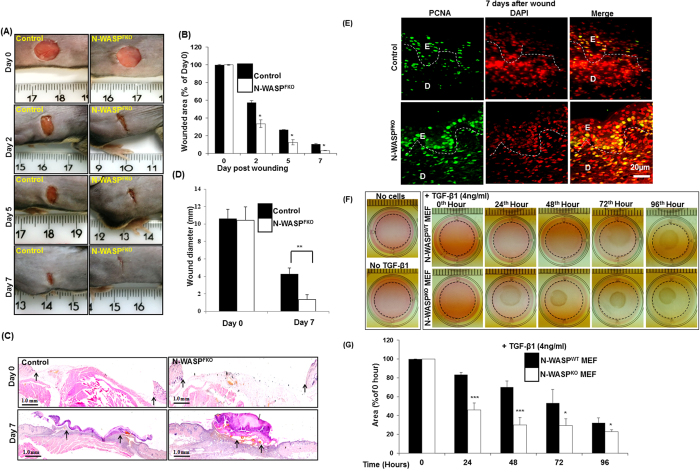
Wound healing is faster in N-WASP^FKO^ mice than control mice. (**A**) Photographs of wound site at different days after wounding (two wounds for each mouse). (**B**) Quantification of wound area at different days after wounding. (**C**) Histomorphometric analysis of wound closure and evaluation of wound closure was performed by measuring the wound diameter of day 0 and day 7 wounds of control versus N-WASP^FKO^ mice (**D**). N-WASP^FKO^ mice showed a significant decrease in wound diameter compared to control mice. (**E**) PCNA staining was performed in order to determine the accelerated wound healing in N-WASP^FKO^ mice. Increased PCNA positive cells were observed at the wound edge as well as in the dermal region in N-WASP^FKO^ mice compared to control mice at 7^th^ day after wound. (**F**) In collagen gel contraction assay, N-WASP^WT^ and N-WASP^KO^ mouse fibroblast cells were embedded in free floating collagen gel in the presence or absence of TGF-β1 (4 ng/ml). Photographs of collagen gels were taken at 24 hour time interval till 96 hours. The boundaries of the wells were outlined with dotted line. (**G**) The areas of collagen gel contracted were quantified using ImageJ and scaled using the ruler taken in the images. N-WASP^KO^ mouse fibroblast cells induced significant faster collagen gel contraction. *p < 0.05; **p < 0.01; ***p < 0.001.

Dermal fibroblasts play a significant role in wound healing and tissue repair^30^. In order to determine the effect of N-WASP deficiency in skin fibroblast on tissue repair, we carried out a wound healing assay on 13–15 weeks old control and N-WASP^FKO^ mice. Mice were subjected to full-thickness excisional wounds and the wounded area of mice were photographed and quantified at indicated time points (Fig. 5). N-WASP^FKO^ mice showed a significant faster wound closure at post-wounding days 2, 5 and 7 compared with control mice (Fig. 5A,B). Moreover, significant reduced wound diameter (shown by arrows) was observed at day 7 in N-WASP^FKO^ mice (Fig. 5C,D). In order to characterize the accelerated wound healing phenotype in N-WASP^FKO^ mice, PCNA staining was performed on skin sections on the 7^th^ day after wounding. We found significantly more number of PCNA positive cells in both dermal and epidermal region in N-WASP^FKO^ mice skin compared to control mice (Fig. 5E). Thus, suggesting that enhanced proliferation rate of dermal/epidermal skin cells after wounding promote faster wound closure in N-WASP^FKO^ mice.

Wound healing involves re-epithelialization as well as wound contraction^31^. Activated keratinocyte proliferate, migrate and subsequently differentiate to form new epithelium while activated fibroblast promote wound healing by wound contraction^2^,^3^,^4^,^5^. In order to characterize the role of N-WASP in wound contraction, we carried out collagen gel contraction assay. We embedded equal number (2 × 10^5^ cells) of WT or N-WASP^KO^ mouse embryonic fibroblasts (MEFs)^25^ in Type I collagen matrix. The collagen gel was detached from the side and stimulated with TGFβ1 to measure gel contraction in free floating collagen gel (Fig. 5F). The gel surface area was measured at 24 hr interval for 96 hours. Collagen gel in the absence of fibroblasts or with cells but without TGFβ1 treatment did not contract (note: a slight collagen gel contraction was observed with N-WASP^KO^ MEF cells even without TGFβ1 (Fig. S3C). In the presence of TGFβ1, collagen gel embedded with N-WASP^KO^ fibroblasts contracted to larger extent compared to embedded with N-WASP^WT^ fibroblasts. (Fig. 5G). Thus, the faster wound healing observed in the N-WASP^FKO^ mice could be due to enhanced wound contraction by the N-WASP deficient fibroblasts.

### N-WASP^FKO^ mice have increased fibroblasts and collagen

Wound healing is a multistep process comprising of, the inflammatory phase, proliferation phase and the maturation phase^32^. In order to determine whether differences in the inflammatory responses could contribute to increased wound healing in N-WASP^FKO^ mice, staining for lymphocytes (CD3/CD4) and neutrophils (Ly-6G antigen) was performed in the granulation tissue. Expression of N-WASP did not significantly alter the ability of wounded tissue to recruit neutrophils, but moderately enhanced the recruitment of lymphocytes (Fig. S4A). Additionally, under basal conditions, the skin of control and N-WASP^FKO^ mice had a very low content of immune cells (data not shown). A cardinal feature of poorly healing wounds is a persistent inflammatory response at the wound site^33^. Despite a slight increase in immune cell infiltration into the wounds of N-WASP^FKO^ mice, the wound closed faster, suggesting that inflammation is unlikely a major contributor to the faster wound healing in N-WASP^FKO^ mouse.

**Figure 6:**
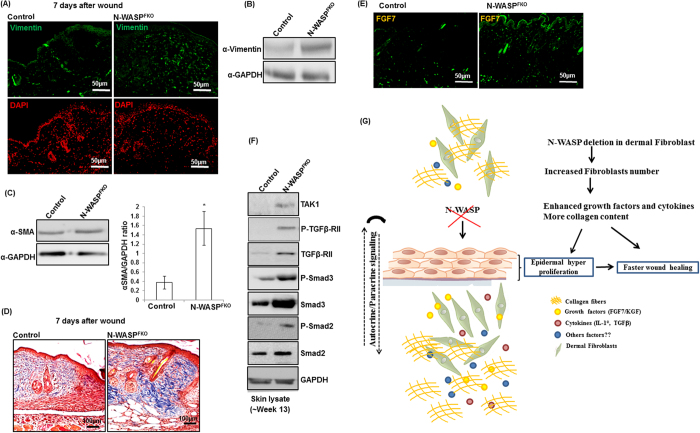
N-WASP^FKO^ mice has increased number of dermal fibroblasts and FGF7 expression. (**A**) Fibroblast recruitment was analyzed by vimentin immunostaining of 7 day after wound. The number of fibroblasts were found elevated in N-WASP^FKO^ mice skin. (**B**) Elevated fibroblast content in N-WASP^FKO^ mice skin was confirmed by western blot for vimentin. (**C**) Western blot analysis for α-SMA from whole skin lysate of control and N-WASP^FKO^ mice. N-WASP^FKO^ mice showed enhanced α-SMA expression in skin compared with control mice. (**D**) Collagen content (7 day after wound) was determined by staining with masson’s trichrome and found to more in N-WASP^FKO^ mice. (**E**) Staining with FGF7 antibody, showed elevated expression in N-WASP^FKO^ mice skin. (**F**) Western blot analysis for TGFβ receptor signaling in the control and N-WASP^FKO^ mice skin. Western blot analysis using GAPDH mAb confirmed that an equal amount of protein was loaded onto each lane. Representative results from three individual animals in each group are shown here. (**G**) Representative observations of this study: N-WASP deletion in dermal fibroblasts led to enhanced proliferation probably by activation of autocrine/paracrine signaling, which caused increase in secretion/expression of growth factors/cytokines. Thus, resulted in epidermal hyperproliferation and accelerated wound healing.

Fibroblasts have been shown to accumulate at the wounded tissue and promote re-epithelialization by producing ECM (collagen) and growth factors^34^. Although Vimentin is expressed by endothelial cells, it can still be used to identify fibroblast^35^,^36^,^37^. Immunohistochemistry analysis revealed that N-WASP^FKO^ mice wound had a significant increase in fibroblast number compared to control mice (Fig. 6A). In addition, Western blot analysis also showed increased vimentin expression in N-WASP^FKO^ mice skin (Fig. 6B). Moreover, a moderate increase in vimentin staining was observed in normal N-WASP^FKO^ mice skin (without wounded) compared to control mice skin (Fig. S3A). Dermal fibroblast cells can also differentiate into myofibroblasts and can promote wound healing. Therefore, we analyzed the effect of N-WASP deletion on myofibroblast conversion. N-WASP^FKO^ mice skin showed significant enhanced expression of α-SMA compared to control mice indicating that N-WASP deletion in dermal fibroblast affect myofibroblast differentiation (Fig. 6C). We further evaluated the amount of matrix deposition (collagen content) by Masson’s trichrome staining. A moderate increase in collagen content in 7 day wound section was observed in N-WASP^FKO^ mice compared to control mice (Fig. 6D). Intense collagen staining was also observed in N-WASP^FKO^ mice skin (non-wounded) (Fig. S3B). This could be due to the increased number of dermal fibroblast in N-WASP^FKO^ mice skin.

### TGFβ signaling is increased in N-WASP^FKO^ mice skin

The higher collagen content in the granulation tissue of N-WASP^FKO^ mice suggested the possibility of increased in growth factor secretion. Moreover, crosstalk between fibroblast and keratinocyte occurs through secreted growth factors are essential during wound healing. Members of fibroblast growth factor family (FGFs) have a broad mitogenic spectrum except FGF7/KGF, which is a specific mitogen for epithelial cells^38^. Analysis of FGF7 expression revealed increase in FGF7 staining in N-WASP^FKO^ mice skin (Fig. 6E). Moreover, moderate increase in pro-inflammatory cytokine IL-1α was also observed in N-WASP^FKO^ mice skin (Fig. S4B). N-WASP expression in keratinocytes has been shown to regulate TGFβ signaling^28^. Based on this information, we analyzed TGFβ signaling using skin lysate from control and N-WASP^FKO^ mice. N-WASP^FKO^ mice expressed higher levels of TGFβ-RII, Samd2/3, TAK1, and their respective activated forms (p-Samd2/3, pTGFβ-RII) (Fig. 6F, S4C). Our results suggest that deletion of N-WASP expression in dermal fibroblast leads to increase in fibroblasts number, leading to increase in collagen content and secretion of growth factors/cytokines/enhanced TGFβ signaling which results in epidermal hyperproliferation and faster wound healing in N-WASP^FKO^ mice.

## Discussion

N-WASP, a member of the WASP family of proteins, regulates the actin polymerization by regulating the activity of the Arp2/3 complex^14^. N-WASP is expressed ubiquitously and N-WASP knockout mice were found to be embryonically lethal suggesting that N-WASP is an essential gene in during the development of mice^24^,^25^. The N-WASP knockout embryos were found to survive past gastrulation stage and initiate organogenesis but die before embryonic day 12 with neural tube and cardiac defects^25^. Thus a number of studies have used the *lox*P-Cre system to generate conditional N-WASP knockout mice to study the role of N-WASP in various tissues. Conditional knockout of N-WASP in muscles using Myf5-cre and MyoD-cre resulted in post-natal death^39^. Similarly, conditional knockout of N-WASP in brain using Nestin-cre also resulted in post-natal death probably due to hydrocephalus^26^. Two research groups have generated conditional knockout mice with expression of N-WASP ablated in keratinocytes using K5-cre^28^,^29^, however the two groups reported contrasting roles for N-WASP in keratinocytes. Leferver *et al*. showed that lack N-WASP expression in keratinocytes caused reduced growth of the keratinocytes while Lyubimova *et al*. showed that N-WASP deficiency leads to epidermal hyper proliferation^28^,^29^.

We have previously found that N-WASP in fibroblasts plays a critical role in cell-ECM adhesion^15^. In order to characterize the role of N-WASP in fibroblasts in the development and maintenance of skin, we generated mice deficient for N-WASP expression in fibroblasts using Fsp-cre as the driver. Mice with the genotype of N-WASP^fl/fl^; Fsp-cre, N-WASP^FKO^ were born following Mendelian genetics, and did not show any abnormalities up to 1 year of growth in the laboratory. There were neither physical abnormalities nor reduced sexual vigor observed in N-WASP^FKO^ mice compared with controls (Data not shown).

Histological sections of the mice skin showed that the N-WASP^FKO^ mice had a thicker epidermis compared with controls and immunostaining for PCNA suggested that the keratinocytes in N-WASP^FKO^ mice were undergoing hyperproliferation (Fig. 2). The fibroblasts and keratinocytes in the skin have a complex interaction in order to maintain homeostasis of the skin and are very critical during wound healing^31^,^40^. Thus we characterized the wound healing characteristics of the N-WASP^FKO^ mice and found that N-WASP^FKO^ mice healed the wound faster than that of control mice, the difference was significant at all the time points analyzed (Fig. 5A–D). Conditional knock out N-WASP in keratinocytes led to hyperproliferation however there was no difference in the wound healing characteristics of the knockout mice compared to the control^29^. Thus the hyperproliferation of keratinocytes seen in N-WASP^FKO^ is probably not the sole reason for faster wound healing in N-WASP^FKO^. Upon injury the fibroblasts promotes wound healing by several mechanisms^31^,^41^, the fibroblasts proliferate, synthesis collagen and differentiate to myofibroblasts which helps in wound healing by contracting the wound^31^,^42^. Thus we analyzed the collagen gel contraction properties of WT and N-WASP deficient fibroblasts by TGFβ treatment of cells embedded in collagen matrix. The N-WASP deficient fibroblasts induced a larger gel contraction compared with WT fibroblasts (Fig. 5G). Notably, our finding was in contrast to a previous *in vitro* study that suggested a role of N-WASP in integrating FAK and Arp2/3 signaling to mediate formation of α-SMA-containing cytoplasmic filaments during myofibroblast differentiation and maturation^43^. In that study, fibroblasts with decreased N-WASP expression (N-WASP knockdown) showed impaired ability to contract collagen gels in response to TGF-β1^43^. The reason for this discrepancy could be due to the difference between the type of cells used. We used knockout cells while Cai *et al*., used knockdown cells. A number of studies have shown that knockout cells may have different phenotypes to knockdown cells; caveolin-1 knockout cells had higher proliferation than control WT cells while caveolin-1 knockdown (80% reduction) did not exhibit increased proliferation^44^. Expression of N-WASP was found to be higher in chronic wound compared to acute wound^45^. HaCaT cells (keratinocytes) treated with N-WASP inhibitors (Wiskostatin or 178-1) had increased cell migration. Similarly, knocking down N-WASP expression also caused increased cell migration^45^. Both the chemical inhibitors were found to enhance wound healing in mice, suggesting that inhibition of N-WASP promotes wound healing^45^. The inhibitors are likely to inhibit N-WASP in all cell types thus it is not possible to identify the cell in which N-WASP activity has to be modulated to promote wound healing. Our studies are consistent with the finding that N-WASP inhibition promotes wound healing.

Various growth factors such as fibroblast growth factors (FGFs), platelet derived growth factors (PDGFs) and cytokines play critical roles during wound healing^38^. PDGFs have been shown to enhance the fibroblast cells proliferation, extracellular matrix production and stimulates fibroblast contraction in collagen matrices^46^. Moreover, impaired wound healing in aged mice is associated with delay in appearance of PDGF receptors^47^. In our study, N-WASP^FKO^ mice skin had increased number of fibroblast compared to control mice as determined by elevated expression of vimentin and increased number vimentin positive cells (Fig. 6A,B). The increased number of fibroblast probably led to enhanced deposition of collagen in N-WASP^FKO^ mice wounded skin tissue (Fig. 6D). A moderate but not significant increase in PDGFR was also observed in N-WASP^FKO^ mice skin (Data not shown). Among FGF family members, FGF7 has been shown to specifically target keratinocytes. FGF7 is secreted by dermal fibroblast and its expression is elevated by injury to skin^38^. Previous reports suggest that FGF7 induces epidermal cell hyperproliferation and migration without affecting their final differentiation or barrier function, and thus promoted faster healing of wounds^48^. In addition, epidermal atrophy with dermal thickening and delay in re-epithelialization of wounds has been reported in transgenic mice expressing dominant negative FGF7 receptor^49^. Consistent with previous studies, expression of FGF7 was found to be elevated in N-WASP^FKO^ mice skin compared to control mice (Fig. 6E) which probably caused epidermal hyperproliferation (without altering epidermal cell differentiation and barrier function) (Fig. 3) and faster wound healing. Moreover, expression of inflammatory cytokines IL-1α was found to be upregulated in N-WASP^FKO^ mice skin (Fig. S4B). IL-1α is secreted by various kinds of cells such as neutrophils, macrophages and fibroblasts. It is very likely that IL-1α stimulates keratinocytes and fibroblast proliferation and regulates wound healing as tropical application of IL-1α accelerated epidermal wound healing^50^. The relationship between IL-1α and FGF7 underscores an important epithelial-mesenchymal communication for skin homeostasis. Keratinocytes are known to synthesize IL-1α in response to injury and IL-1α has been shown to stimulate fibroblast and keratinocyte growth, collagen synthesis by fibroblasts, and chemotaxis of keratinocytes^51^,^52^. In a double paracrine manner, keratinocyte IL-1α stimulates the expression FGF7 in fibroblasts^53^,^54^. The fibroblast FGF7 enhances epidermal proliferation. The deletion of N-WASP in the fibroblast perturbed this epithelial-mesenchymal communication, which manifested as epidermal hyperproliferation and increased fibroblast number.

Fibroblasts produce many components of ECM and are also a relevant source of TGFβ^55^. Our results suggest that N-WASP modulates TGFβ signaling in fibroblasts as we found enhanced expression of TGFβ signaling proteins in N-WASP^FKO^ mice skin (Fig. 6F, S4C) and N-WASP^KO^ MEFs caused a slight but significant *in-vitro* collagen gel contraction even in the absence of exogenous TGFβ (Fig. S3C). Both WT MEFs and N-WASP^KO^ MEF caused *in-vitro* collagen gel contraction in the presence of exogenous application of TGFβ however the N-WASP^KO^ MEF caused significantly higher gel contraction (Fig. 5G). TGFβ signal through binding to two TGF-β receptors (TGFβ-RI and TGFβ-RII). TGFβ-RII is constitutively phosphorylated and on binding of the ligand, phosphorylates TGFβ-RI. Phosphorylation of the receptor complex activates the SMAD intracellular signaling pathway through the receptor Smads (Smad-2 and Smad-3) and co-Smad 4. The receptor SMADs and Smad-4 cross over the nuclear membrane where they regulate a number of genes^56^. In N-WASP^FKO^ mice skin, expression of Smad-2 and Smad-3 and respective phospho-Smad2 and phospho-Smad-3 were elevated (note: we do not observe any difference in ratio of p-Samd3/Samd3 or p-TGFβ-RII/TGFβ-RII between control and N-WASP^FKO^ mice skin, thus enhanced expression of these TGFβ signaling associated factors is probably causing the significant differences in the wound healing between control and N-WASP^FKO^ mice) (Fig. S4C). This enhanced expression of Smad-2 might have up-regulated the expression of receptor TGFβ-RII via positive feedback loop and thus representing the increased TGFβ signaling in N-WASP^FKO^ mice skin^56^. TGFβ is a mediator of tissue repair, wound healing and has been widely implicated in progressive tissue fibrosis^57^. TGFβ drives fibroblast-myofibroblast differentiation. Myofibroblasts express α-smooth muscle actin (α-SMA) and are present in granulation tissue where they are responsible for wound contraction. TGFβ has been shown to upregulate the expression of α-SMA both *in vitro* and *in vivo*^58^,^59^. Conceivably, the enhanced TGFβ signaling stimulates fibroblast proliferation and also activates normal fibroblasts to become myofibroblasts. A recent study suggests that reconstitution of vimentin expression in vimentin^−/−^ fibroblasts restored their proliferation and TGFβ production, thus is critical for wound healing^60^. This study further potentiates our observation; enhanced vimentin expression/enhanced fibroblasts proliferation/enhanced TGFβ signaling. The enhanced TGFβ signaling associated factors in N-WASP^FKO^ mice skin probably promotes fibroblasts to myofibroblasts differentiation and faster wound healing. Taken together; our observations suggest that ablation of N-WASP expression in dermal fibroblasts, increase fibroblast cell number, which is probably due to the activation of fibroblasts by autocrine/paracrine signaling. This activation of fibroblasts in N-WASP^FKO^ mice, enhanced expression/secretion of growth factors/cytokine (FGF7/IL-1α/TGFβ signaling) or collagen deposition, resulted in epidermal hyperproliferation and accelerated wound healing in aged mice (Fig. 6G).

## Materials and Methods

### Animals

*N-WASP*^*fl/fl*^ mice were generated as described previously^26^. In order to generate mouse skin fibroblast cells specific N-WASP expression deletion, *N-WASP*^*fl/fl*^ mice were crossed with Fsp-cre (fibroblast specific protein) mice followed by back cross. Genotyping of mice were performed as described previously^26^. In brief, small piece of tail tips (2–5 mm) were digested in tail digestion buffer containing 50 mMKCl, 10 mMTris–HCl (pH 9.0), 0.1% Triton X-100, and 0.4 mg/mL Proteinase K (Sigma-Aldrich; P2308). Tail PCR was performed using KAPA HiFi PCR Kit (KK2101). The primers used to detect flox/flox were (5′ to 3′ direction); AGCTCAGAGAAGGTGTATTGG (forward) and AGGA CTTACATCTCCAGCAAAGG (reverse). Fsp-cre primers used were: GCGGTC TGGCAGTAAAAACTATC (forward), GTGAAACAGCATTGCTGTCACTT (reverse); internal control primers: CTAGGCCACAGAATTGAAAGATC-3′ (forward), GTAGGT GGAAATTCTAGCATCAT (reverse). In order to detect the deletion of N-WASP at genomic level, another reserve primer (WASL-Flr) has been used together with flox/flox 5′ primer; GGACAGGGTCTATCTCTGAATTCCT. *N-WASP*^*fl/fl;*^*Fsp-cre* (N-WASP^FKO^) and control (*N-WASP*^*fl/fl*^) mice were maintained on standard chow diet with unlimited water at constant temperature of 20 °C and 12/12 hours of artificial dark/light cycle. All the experiments were performed according to approved protocols of Institutional Animal Care and Use Committee. All the animal experiments performed were approved by Institutional Animal Care and Use Committee (IACUC) of Nanyang Technological University (NTU), (ARF-SBS/NIE-A0250 and ARF-SBS/NIE-A0236).

### Histology

Mice were anaesthetized and sacrificed via cervical dislocation. Dorsal skin of mice was flattened on parafilm and fixed in 4% paraformaldehyde overnight in 4 °C. Fixed dorsal skin tissue was then washed with 1X PBS and dehydrated using a different percentage of absolute ethanol solution (70, 80, 90 and 100%) for 1 hour each followed by dehydration in 50% xylene/ethanol mixture and 100% xylene solution. Dehydrated samples were then submerged in paraffin wax at 60 °C overnight and subsequently embedded in paraffin molds. Paraffin embedded tissue was sectioned to 5 μm size on superfrost slides (Fisher). For staining, slides were placed in 60 °C to dissolve the paraffin and subsequently rehydrated and stained with haematoxylin and eosin. Sections were stained with respective primary antibodies; anti-E-cadherin (BD Bioscience; 610181), keratin 10 (sc-23877), involucrin (sc-28558), transglutaminase (sc-25786), KGF7 (sc-1365) and vimentin (550513, BD Biosciences). Fluorescence labeled secondary antibodies (Alexa-488 or 594; Thermo Fisher Scientific) were used at 1:100 dilution. For analysis of proliferating cells, PCNA antibody (sc-56; PC10) was used to stained paraffin embedded dorsal skin sections. Quantification of PCNA positive cells was performed using ImageJ software^61^. PCNA positive cells were counted as number of PCNA positive cells per microscopic field. Images were taken using the 40X objective and minimum 6 microscopic fields were counted for each sample for individual experiment and experiment was repeated thrice. Fluorescent images were captured using Olympus microscope fitted with CoolSNAP^HQ2^ camera.

### Wound healing assay

13–15 weeks old control (n = 16) and N-WASP^FKO^ male mice (n = 16) were anesthetized using Ketamine/Xylazine (90 μg/10 μg). Mice were shaved at dorsal side and cleaned with alcohol. Two full thickness circular, (average size of 10 mm in diameter) wounds were made per mouse (separated by at least 1 cm of uninjured skin) using forceps and scissor at dorsal side without injuring the underlying muscle as described^62^,^63^. The wounds were covered with occlusive dressing (Tegaderm; 3M^TM^) in order to prevent any infection. The wounds were photographed with scale using digital camera at 0, 2, 5 and 7^th^ day post injury. The wounded area was determined using imageJ software and wound closure rate was determined as percentage of wound area to the initial wound size. Skin biopsies for histology were performed from each wound at day 0 and 7 after surgery. To visualize collagen, skin sections were stained with masson’s trichrome (Cat No: 25088A-F, Polysciences Inc).

### TPA induced hyperproliferation

13–15 weeks old control and N-WASP^FKO^ mice were shaved 24 hours before TPA application (6.5 nM/50 μl in solvent (acetone)) on the dorsal skin. Mice were sacrificed after 24 hours of TPA treatment; skin was isolated, fixed in 4% paraformaldehyde/PBS at 4 °C overnight and embedded in paraffin for histological sections. Sections were stained with haematoxylin and eosin, PCNA and E-cadherin antibodies.

### Lucifer yellow penetration assay

For Lucifer yellow penetration assay, 1 mM of Lucifer yellow was poured on to the 1 cm^2^ piece of skin tissue (dorsal skin, epidermal side) with precautions taken as dye was not allowed to go through the dermal side of the skin. After incubation for 1 hour at 37 °C, the skin was washed excessively with PBS and skin was cryo-sectioned. Cryo-sections were stained with DAPI and analyzed with fluorescence microscope.

### Collagen gel contraction assay

Experiment was performed as described previously with some modifications^64^. N-WASP^WT^ and N-WASP^KO^ mouse embryonic fibroblast cells were maintained in DMEM/10% FBS media. N-WASP^WT^ and N-WASP^KO^ MEFs were kind gift from Scott B Snapper lab^25^. Mouse embryonic fibroblast cells were trypsinised, counted and 200,000 cells were added into 0.5 mg/ml of Corning Rat Tail Collagen I solution (Corning, USA). 1 μM NaOH was immediately added to neutralize the mixture. The entire mixture was transferred to a 24 well plate immediately. Gel was allowed to set in the 37 °C CO_2_ incubator for three hours. After the gel has solidified, complete DMEM was added. Solidified Collagen Gel was detached from the side of the well. Pre-diluted TGFβ1 was added to achieve a final concentration of 4 ng/ml. The complete media was renewed every 24 hours using complete DMEM containing TGFβ1. Image was taken and quantified using ImageJ every 24 hours starting with the 0 hour image. Images were taken by placing the 24-wells plate, containing the gel, above a light box with a ruler alongside the gel as a standard to calibrate the pixel size.

### Statistical analysis

For statistical significance analysis, unpaired student’s t-test was performed and *p* value < 0.05 was considered as significance. Values in bar charts are the mean ± S.D from three independent experiments.

## Additional Information

**How to cite this article**: Jain, N. *et al*. Conditional knockout of N-WASP in mouse fibroblast caused keratinocyte hyper proliferation and enhanced wound closure. *Sci. Rep.* **6**, 38109; doi: 10.1038/srep38109 (2016).

**Publisher's note:** Springer Nature remains neutral with regard to jurisdictional claims in published maps and institutional affiliations.

## Supplementary Material

Supplementary Information
