# Supplementary material for: Conditional knockout of N-WASP in mouse fibroblast caused keratinocyte hyper proliferation and enhanced wound closure
Source: Sci Rep. 2016 Dec 2;6:38109. doi: 10.1038/srep38109 (PMC5133560; doi:10.1038/srep38109)
Supplement: Supplementary Information [file srep38109-s1.pdf]

Conditional knockout of N-WASP in mouse fibroblast caused keratinocyte hyper proliferation and enhanced wound closure

Neeraj JAIN[1], Pazhanichamy KALAILINGAM[1], Kai Wei TAN[1], Hui Bing TAN[1], Ming Keat SNG[1], Jeremy Soon Kiat CHAN[1], Nguan Soon TAN[1,2,3] and Thirumaran THANABALU[1*]

Figure: S1. Dermal fibroblast cells specific conditional N-WASP knockout mice has normal growth pattern.

(A) Tail genomic PCR genotyping of 1st generation mice. Arrow (↑) indicates heterozygous *N-WASP$^{fl/WT}$; FSP-Cre* mice. **(B)** Back crossed tail genomic PCR genotyping. Asterisk (*) indicates homozygous *N-WASP$^{fl/fl}$; FSP-Cre* mice. **(C)** Tail genomic PCR representing the deletion product of exon 3 and 4 of N-WASP gene. **(D)** Average body weight of represented mice.

Figure: S2. Enhanced epidermal thickness in fibroblast specific N-WASP deficient mice.

(A) H & E stained dorsal skin sections from 5 weeks old mice shoed no defect in hair follicle pattern and number. **(B)** H & E stained dorsal skin sections from 13 weeks old *N-WASP$^{fl/fl}$*, *N-WASP$^{WT/fl}$;FSP-cre* and *N-WASP$^{fl/fl}$;FSP-cre* mice. *N-WASP$^{fl/fl}$;FSP-cre* mice showed significant increase in epidermal thickness.

Figure: S3. Elevated collagen content and collagen contraction by N-WASP^{KO} fibroblast in the absence of TGF-β1

(A). Dermal fibroblast content determined by vimentin staining. N-WASP^{FKO} mice showed enhanced number of fibroblast under normal conditions as well. **(B)**. Collagen content in non-wounded skin (by staining with masson's trichrome) was found to be moderately elevated in N-WASP^{FKO} mice. **(C).** Contraction of collagen by N-WASP^{KO} mouse fibroblast cells even in the absence of added TGF-β1

Figure: S4. Increased infiltration of immune cells and elevated IL-1α in N-WASP^{FKO} mice

(A). Increased infiltration of Immune cells in the skin of N-WASP^{FKO} compared to skin of control Mice. **(B).** Cytokine (IL-1α) expression from whole skin lysate ELISA was found to be significantly up-regulated in N-WASP^{FKO} mice skin. **(C).** Densitometry analysis of TGFβ signaling proteins from normal skin of 13 week old control and N-WASP^{FKO} mice (three mice from each group). Enhanced expression of TGFβ signaling proteins were observed in N-WASP^{FKO} mice.

Supplementary figures

Fig S1

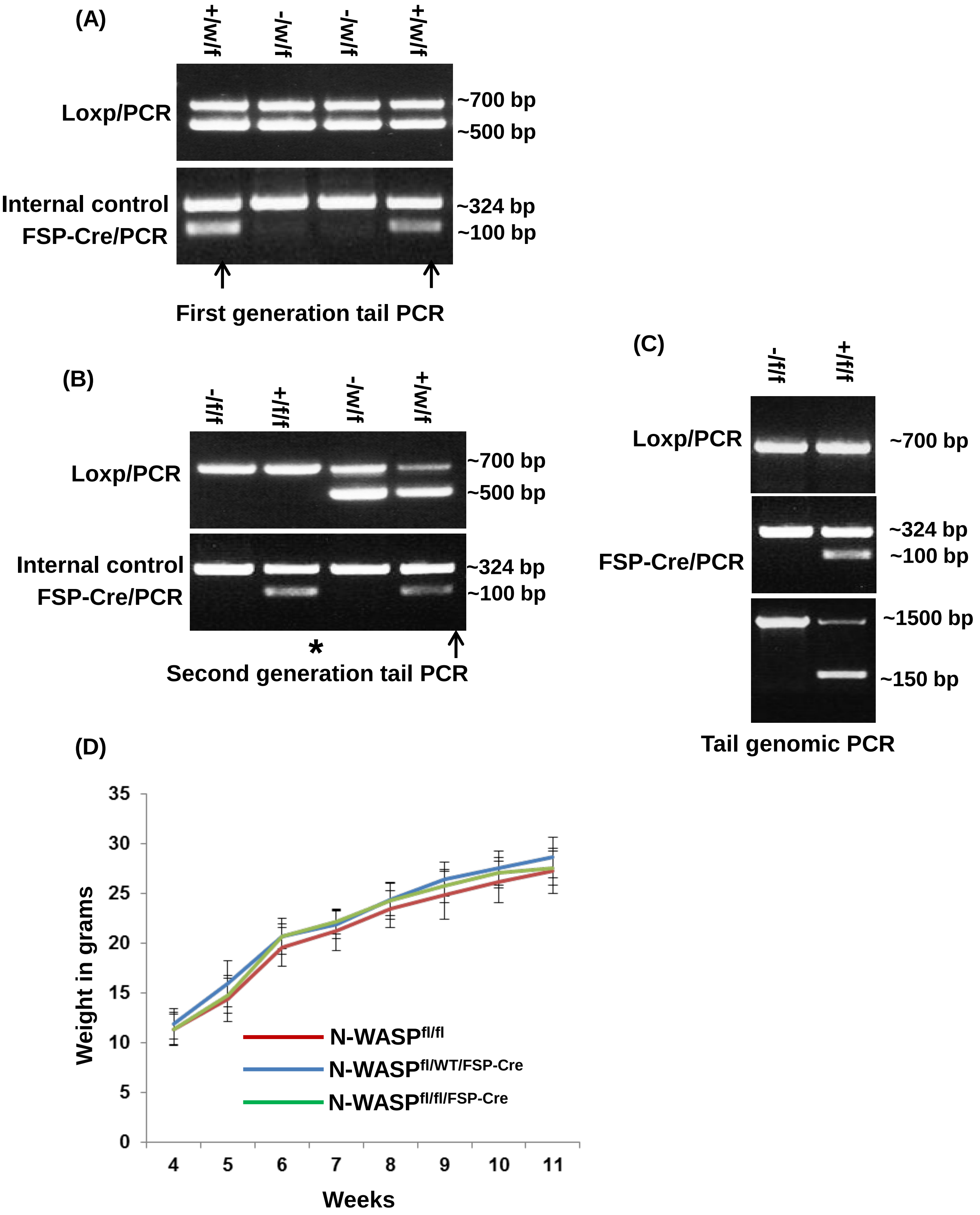

Fig S2

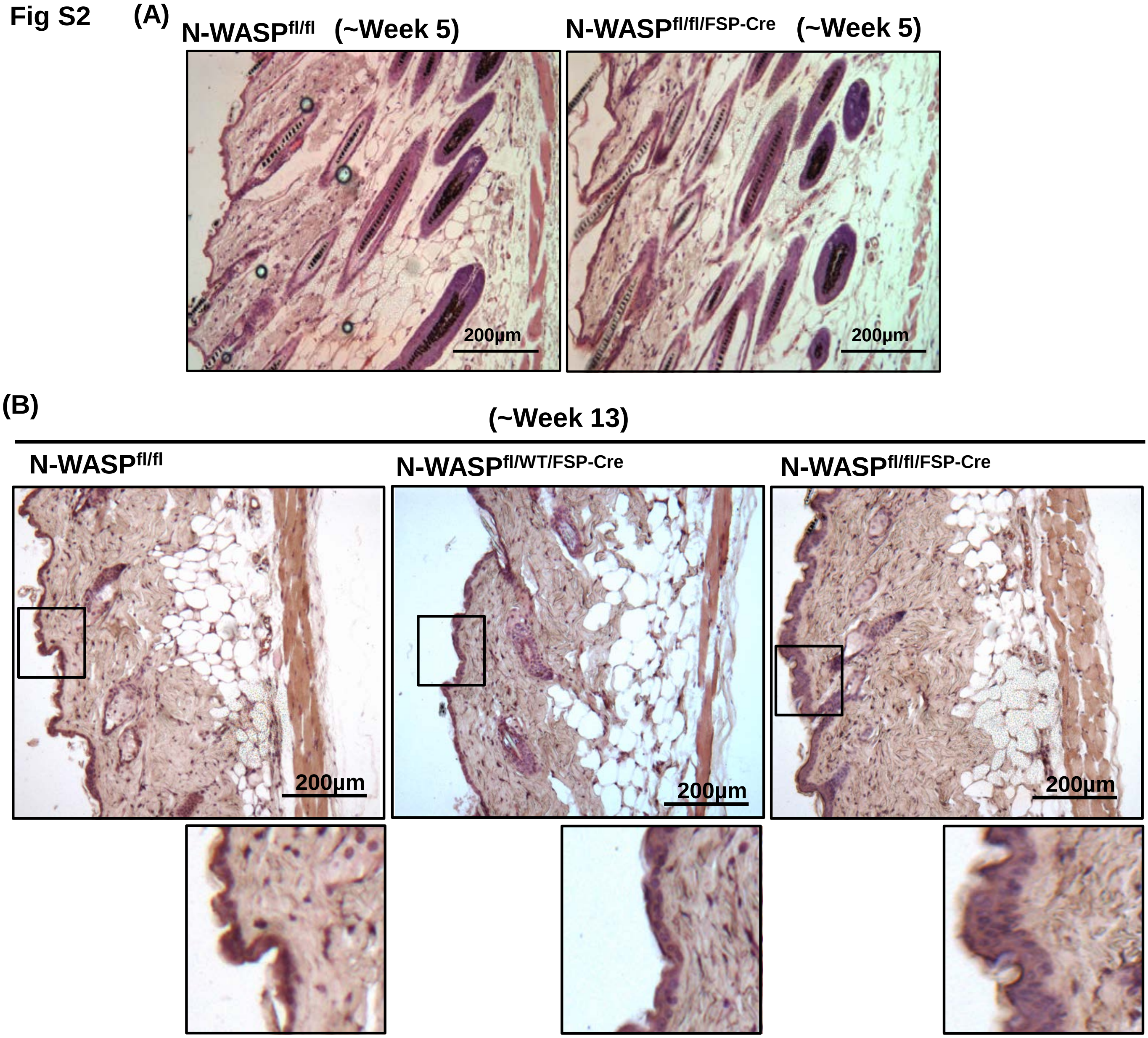
(A)
N-WASP fl/fl (~Week 5)
N-WASP fl/fl/FSP-Cre (~Week 5)
200μm
200μm
(B)
(~Week 13)
N-WASP fl/fl
N-WASP fl/WT/FSP-Cre
N-WASP fl/fl/FSP-Cre
200μm
200μm
200μm

Fig S3

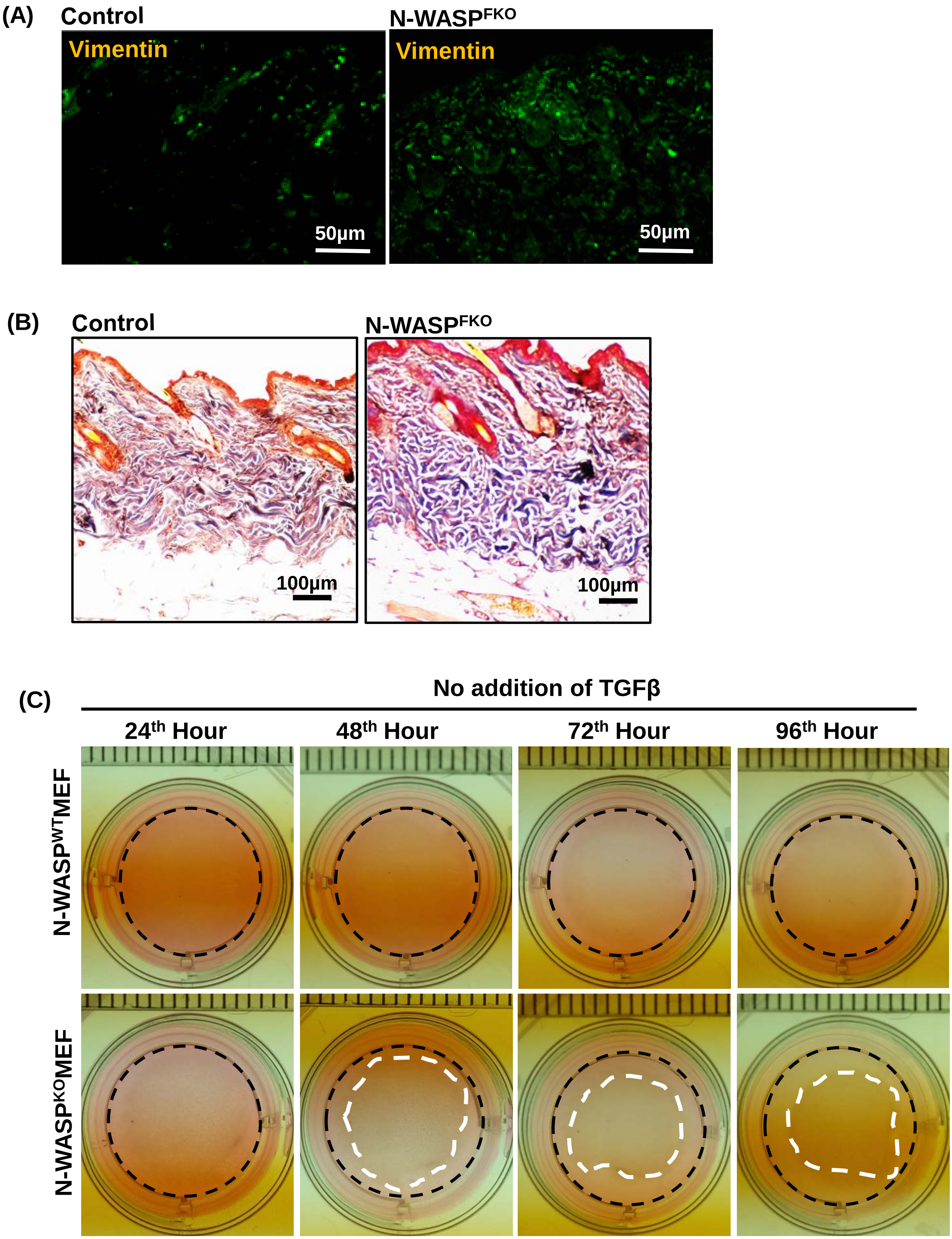
(A)
Control
N-WASPFKO
Vimentin
Vimentin
50μm
50μm
(B)
Control
N-WASPFKO
100μm
100μm
(C)
No addition of TGFβ
24th Hour
48th Hour
72th Hour
96th Hour
N-WASPWTMEF
N-WASPKOMEF

Figure S4

(A)

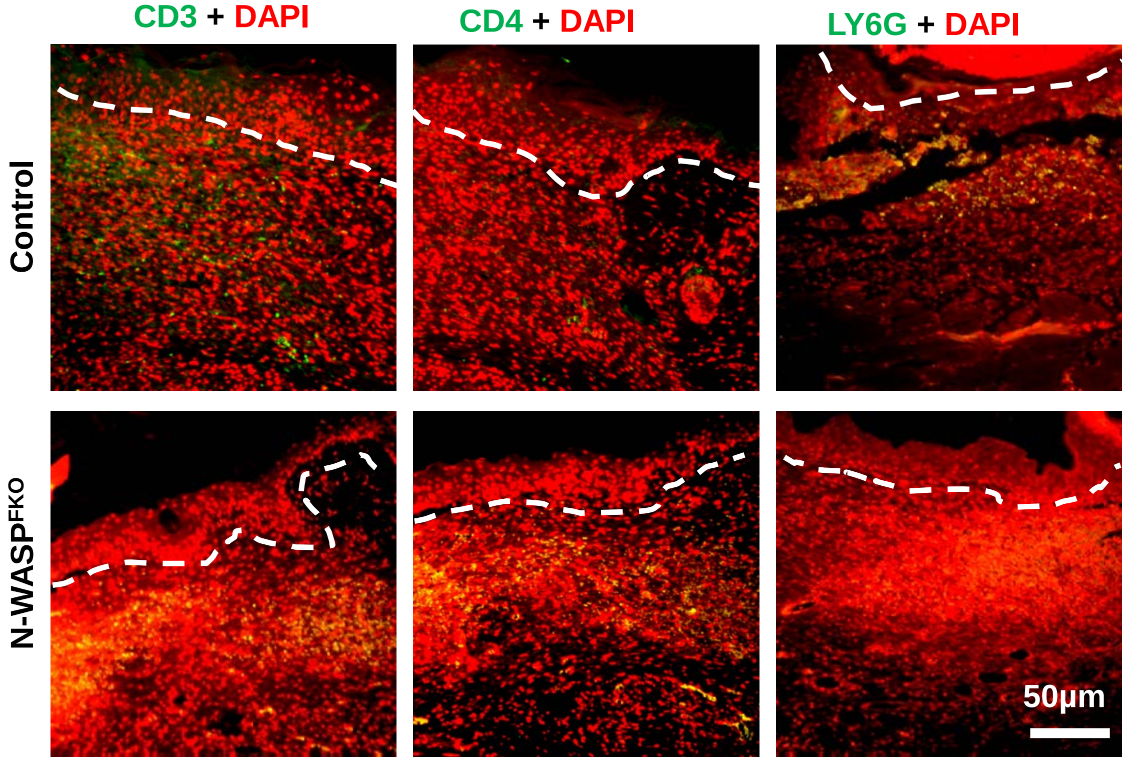
CD3 + DAPI
CD4 + DAPI
LY6G + DAPI
Control
N-WASP^{FKO}
50μm

(B)

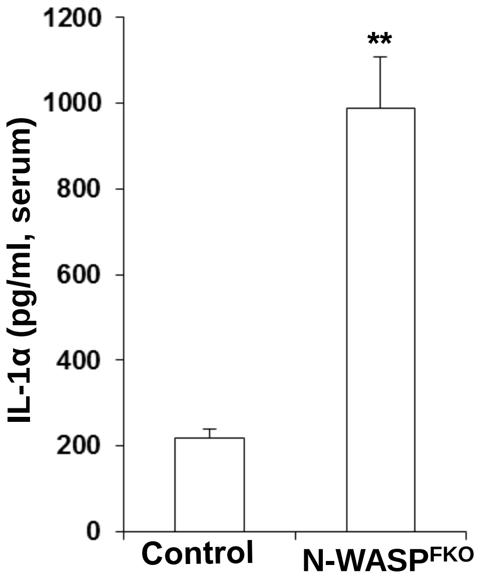
1200
1000
800
600
400
200
0
IL-1α (pg/ml, serum)
**
Control
N-WASP^{FKO}

Figure S4

(C)

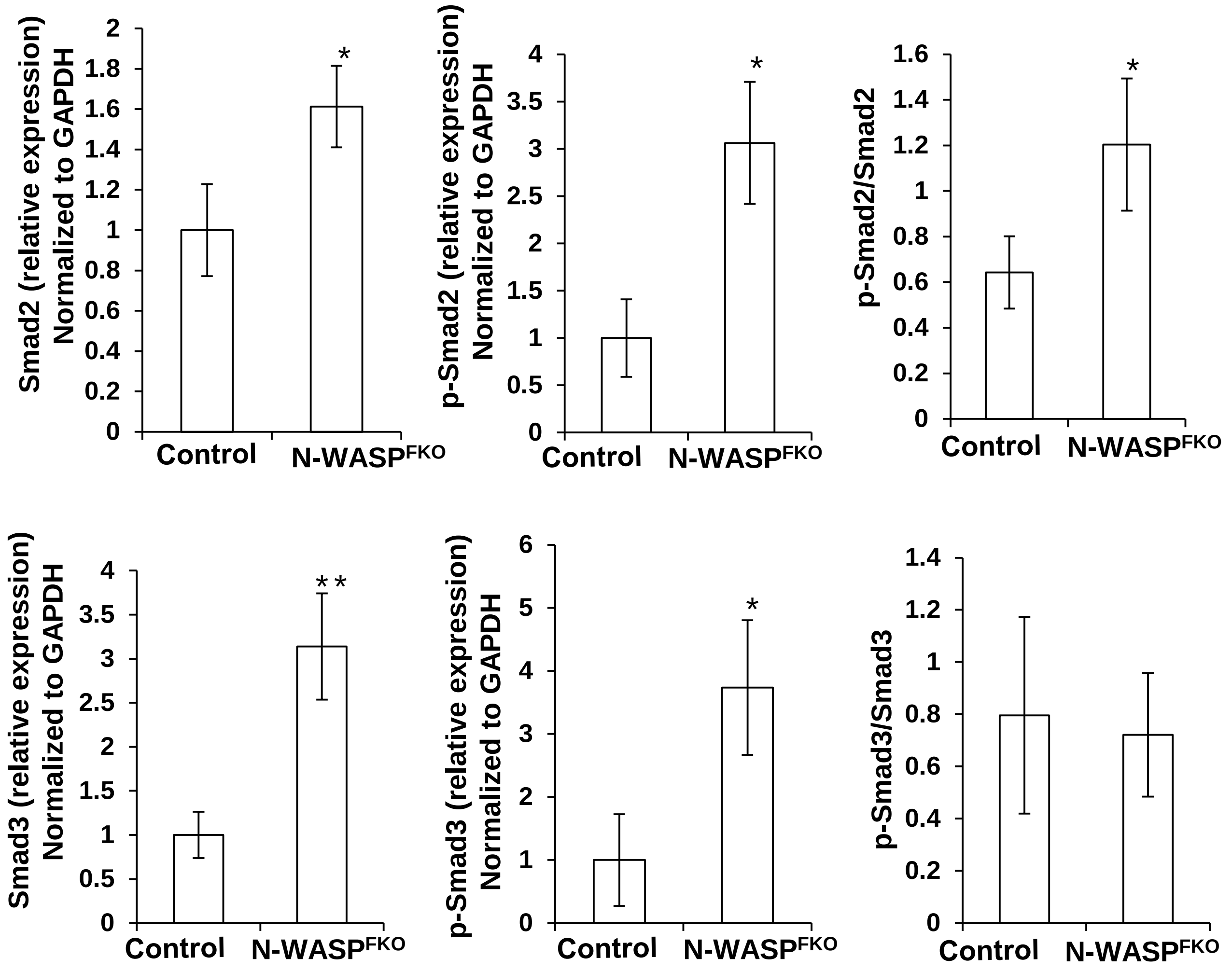

Smad2 (relative expression) Normalized to GAPDH
p-Smad2 (relative expression) Normalized to GAPDH
p-Smad2/Smad2
Smad3 (relative expression) Normalized to GAPDH
p-Smad3 (relative expression) Normalized to GAPDH
p-Smad3/Smad3
Control
N-WASP^FKO
*
**
